# Longitudinal Effects of a sit-stand desk intervention - persistence, Fade-Out, and psychological momentum: a Randomized Controlled Trial

**DOI:** 10.1186/s40359-022-00948-9

**Published:** 2022-11-02

**Authors:** Udo Konradt, Alexander Nath, Sabrina Krys, Frank Heblich

**Affiliations:** 1 Institute of Psychology, Kiel University, Christian-Albrechts-Platz 4, 24118 Kiel, Germany; 2 Corporate Medical Occupational Health Services, Kiel University, Christian-Albrechts-Platz 4, 24118 Kiel, Germany

**Keywords:** Sit-stand desk intervention, Activity-permissive intervention, Health, Velocity, Acceleration, Fade-out

## Abstract

**Background:**

This study examined whether the effects of a sit-stand desk (SSD) intervention on employees’ musculoskeletal complaints (i.e., intensity and prevalence) and activation (i.e., vigilance and vitality) persist or fade out and whether velocity and acceleration of health improvements can predict medium-term (six-month) and long-term (24-month) improvements. Drawing from dynamic models of self-regulation, as well as the psychological momentum theory, we hypothesized that velocity and acceleration of health improvements in the early stages of the intervention would predict medium-term health improvements, which sustain long-term.

**Methods:**

We used data from a six-month seven-wave randomized controlled trial with employees in mostly sedentary occupations and supplemented this by follow-up data from the same participants 18 months later, resulting in eight waves.

**Results:**

Bayesian structural equational modeling revealed no significant intervention effect after 24 months implying a fade-out. But more importantly, velocity and, partially, acceleration of health improvements at earlier stages predicted medium-term improvements in musculoskeletal complaints and long-term improvements in vigilance.

**Conclusion:**

The findings of this study suggest that positive intervention effects fade out over time and health effects benefit from prompt progresses at the beginning of the intervention, warranting exploration in prolonged longitudinal studies.

**Supplementary Information:**

The online version contains supplementary material available at 10.1186/s40359-022-00948-9.

## Introduction

Sedentary work or a lifestyle that involves little or no physical activity has been shown to be a major risk factor for neck pain [1] obesity, metabolic syndrome, abdominal girth [2], and cardiovascular disease [3], as well as for impairments in general health [1, 4]. In occupational settings, activity-permissive workstations and dynamic sitting (e.g., [5]) have thus been proposed as an appropriate way of increasing physical activity and thereby mitigating the harmful effects of prolonged sitting. One approach is to use a sit-stand desk (SSD), which allows employees to alternate between sitting and standing. Although most of the evidence from occupational health research has been deemed to be of only low to moderate quality (for a review, see [6]), evidence suggests that using SSDs reduces neck and back pain and increases vigilance and vitality, two aspects of activation [5, 7–9].

Though there has been some more compelling and higher-quality occupational health research on the effectiveness of SSD interventions [10–12], there are three major gaps that have not yet been sufficiently addressed.

First, the evidence on the beneficial effects of SSDs is limited to relatively short periods of time, lasting from a few hours to 24 weeks, but no previous research has examined the long-term effects (i.e., years). Consequently, we do not know whether SSDs contribute to a sustained improvement in health; it is also not yet clear whether there may be late or lagged effects that occur months or even years after the beginning of any intervention or if effects might fade out. Better understanding is also needed of when and why effects persist, fade out or are stronger in some cases than in others.

Second, previous studies have focused mainly on pathogenic outcomes such as physical complaints and distress and have not looked at potential positive salutogenic effects such as activation, thereby considering only a subset of the potential effects of SSD interventions [5, 13].

Finally, the underlying factors that give rise to change in health are poorly understood. Because an SSD intervention requires employees to change their patterns of behavior, process-oriented models of self-regulating processes during goal pursuit [14, 15] and psychological momentum theory [16–18] provide conceptual frameworks that can help us to understand the effects of an intervention. More precisely, the velocity (i.e., the rate of change in an individual’s health with respect to time; also known as speed) and the acceleration (i.e., the rate of change in velocity) of improvements in people’s health can be decisive for positive affect and satisfaction (e.g., [19, 20]) and thus ultimately for the success of an intervention [21].

This study is the first to address these critical gaps in occupational health research and extends the literature by examining the long-term effects of an SSD intervention on musculoskeletal complaints (i.e., prevalence and intensity) and activation (i.e., vigilance and vitality) in an eight-wave 24-month randomized controlled trial with employees in mostly sedentary occupations. We also aim to identify mechanisms for predicting persistence and preventing fade-out effects of the intervention. Drawing on self-regulation theory and psychological momentum theory, we seek to explore whether the velocity and acceleration of people’s health improvements cause a momentum, which can predict medium- and long-term effects.

### Long-term effects of ssd interventions

SSD interventions—the implementation of height-adjustable desks—allow people to avoid unbalanced posture and to increase their overall activity during their work by alternating between sitting and standing. Extensive research has shown that prolonged sitting is a risk factor for musculoskeletal complaints (MSC) in the back, neck and shoulders (see [1, 4, 9] for reviews). MSC are particularly important in occupational contexts because they are strongly affected by ergonomic conditions and a main reason for physical disability at work and long-term absenteeism [22], and are associated with reduced work performance and work ability [23]. Consequently, workplace risk management strategies and workplace interventions have been considered crucial for reducing these risks.

Conclusions drawn from high-quality studies suggest that SSDs are an effective way to reduce both prevalence and intensity of MSC in employees. Gao and colleagues [10] demonstrated that those who took part in an SSD intervention reported significantly less musculoskeletal discomfort in the neck and shoulders six months later. Likewise, Pronk et al. [24] found that SSDs reduced upper back and neck pain by 54% approximately two months later. Moreira-Silva et al. [7] provided similar findings in their meta-analysis. Konradt et al. [12] found moderate to high inhibiting effects on the intensity and prevalence of MSC after six months of SSD use, both at the within- and between-person level. SSD interventions are thus effective in reducing people’s MSC immediately and in the medium term (i.e., months after intervention). However, it is not yet clear whether the intervention effects persist, diminish or even disappear over longer observation periods, such as years. Evidence from educational interventions suggest that fade-out is a widespread and substantive phenomenon (see [25], for a review).

Activity-permissive interventions in occupational settings, such as SSDs, are expected to support employees in standing or light physical activity and possibly change their beliefs and habits of the target behavior. As we have no evidence that the reported medium-term intervention effect (e.g., [10–12]) disappears or that there are fade-out effects, we assume that the effects persist in the longer term.

#### Hypothesis 1

Employees in the SSD intervention group show a long-term (i.e., 24 months after the baseline) decrease in prevalence and intensity of MSC compared to employees in the control group.

The use of SSDs is also expected to improve users’ psychological health, including their perceived activation, which refers to mental and physical energy and the state of being attentive and responsive [26, 27]. In a randomized controlled study on SSDs, Dutta et al. [28] observed that employees using SDDs felt more energized and less tired. Mailey et al. [29] found that regularly alternating between sitting and standing (i.e., once every half hour) increased people’s perceived energy and led to less tiredness compared to taking two 15-minute breaks during each working day. Drawing on Hobi’s [26] two-dimensional concept of activation, Konradt et al. [12] revealed that employees’ perceptions of activation (i.e., vitality and vigilance) improved significantly across six months of SSD use. Yet, however, it is not clear whether an SSD intervention also has long-term effects on people’s activation level or whether the effects fade out. We thus hypothesize:

#### Hypothesis 2

Employees in the SSD intervention group show a long-term (i.e., 24 months after the baseline) increase in activation (i.e., vitality and vigilance) compared to employees in the control group.

### Velocity and acceleration of improvement and psychological momentum

Health intervention effects have been construed as cognitive, affective, and behavioral self-regulation processes by which individuals monitor and adapt their goal-relevant behavior [30]. Self-regulation theory [14, 31] provides a framework for describing “the processes involved in attaining and maintaining (i.e., keeping regular) goals, where goals are internally represented (i.e., within the self) desired states” ([32], p. 158). The theory suggests that the amount of attention and effort individuals put into attaining their goals is affected not only by how far they are from their goal (i.e., the discrepancy between their current state and the goal state) but also by how they make progress towards it, i.e. how quickly that discrepancy decreases (i.e., velocity). The process-oriented model of self-regulation [15] defines progress as the change in the value or magnitude of a variable over a defined period of time where the direction of change is considered positive. Velocity is defined as “the rate at which discrepancies between current and goal states are changing” ([15], p. 63). Johnson and colleagues argue that the perception of high velocity in attaining a goal has the strongest effect in terms of increasing the motivation and ability to keep up a sustained effort. In cases where there is little or no velocity in attaining their goals, individuals might revise their goal downward over time, which should result in behavioral disengagement.

Psychological momentum (PM) theory [16–18] provides another conceptual framework for understanding the psychological mechanisms that may facilitate or inhibit healthy behavioral practices. PM is based on the notion that future behavior is more likely to be consistent with past behavior, because this earlier behavior has unleashed a psychological force or impulse that effects performance. Briki and Markman [33] defined PM as the “cognitive, emotional, physiological, and behavioral responses to perceptions of progress moving either toward or away from a goal state” ([33], p. 2) and construed PM as the phenomenological experience of goal pursuit. PM theory proposes that the velocity of progress, conceptualized as the direction and magnitude of a change towards a certain goal [34], leads to positive or negative momentum.

Empirical research provides support for the notion that velocity plays a significant role in progress towards a goal. Individuals typically prefer to work towards goals that can be achieved more quickly [35], and where discrepancies between the current state and the goal state are more short-lived [19, 20]. Woolley and Fishbach [21] found evidence that persistence in goal-related behavior (e.g., changing one’s behavior to eliminate unhealthy habits) was greater when the rewards were more immediate. Chang et al. [36] also found that individuals set their goals lower when they experienced little or no velocity in achieving those goals. Higher velocity was also found to be positively related to affective reactions to goal pursuit, such as better mood [37], increased positive affect and decreased negative affect [36, 38], and higher levels of satisfaction [36]. Research in organizational settings suggests several functional forms of physical or psychological health trajectories (e.g., [12, 39]). However, taking velocity of improvement into account offers a more detailed and comprehensive picture how occupational interventions work. Self-regulation theory and research suggest that fast velocity in health improvement leads to higher persistence in health-promoting behavior.

#### Hypothesis

**a**: Employees in the SSD intervention group with initially high velocity of decrease in MSC show (a) lower medium-term and (b) lower long-term levels of MSC.

#### Hypothesis

**b**: Employees in the SSD intervention group with initially high velocity of increase in activation show (a) higher medium-term and (b) higher long-term levels of activation.

Acceleration also plays a role in individuals’ pursuit of a goal [15, 40]. It represents the change in velocity over time, that is the change in current velocity relative to past velocity (the velocity can speed up or slow down). Carver and Scheier [14, 31] argued that an increase in velocity improvement—that is acceleration—leads to a shift toward higher levels of positive affect and satisfaction. Pertaining research in clinical settings suggests that an “accelerated treatment response is arguably a more efficient treatment; not only getting better, but getting better faster” ([41], p. 1117). However, we are unaware of any efforts to study the role of acceleration in occupational health interventions. Therefore, our goal is to develop an initial understanding of the role of acceleration.

#### Hypothesis

**a**: Employees in the SSD intervention group with initially high acceleration of decrease in MSC show (a) lower medium-term and (b) lower long-term levels of MSC.

#### Hypothesis

**b**: Employees in the SSD intervention group with initially high acceleration of increase in activation show (a) higher medium-term and (b) higher long-term levels of activation.

## Method

### Sample and design

The present ancillary study used data from a longitudinal randomized controlled trial field study by Konradt et al. [12], with new data being collected and derived to answer questions that were not part of the primary study.

The primary study’s main goal was the investigation of health promoting spiraling effects over the course of six months. Physical and psychovegetative complaints as well as positive (activation) and negative (stressor uncontrollability) psychological symptoms over the course of six month were recorded. Using latent growth curve analyses as well as cross-lagged-panel modelling in a Bayesian setting the authors found inhibiting effects for pathogenetic outcomes as well as promoting effects for positive indicators. However, no change in stressor uncontrollability was found. The current ancillary study aims to investigate the persistence of the effects in musculoskeletal complaints and activation 18 months after the primary study’s end. Furthermore, earlier changes in symptomatology versus assessment of overall progression will be brought to the forefront and examined in more detail. The sample described in the subsequent paragraphs is the same for both the primary as well as the ancillary study regarding the first seven measurement occasions (T0-T6).

The study was conducted between 2015 and 2017 at < blinded >. In the primary study, 127 employees in various, mostly sedentary, occupations were randomly assigned to either an intervention or a control group by block randomization, resulting in an equal number of assignments to each group. The random allocation procedure was prepared and conducted by the last author and a student assistant. The other authors were blinded, but participants were not blinded qua design. The organization, comprised of a mix of 3,500 employees, engaged in a wide variety of occupations including administration, maintenance and repair, IT, R&D, and teaching.

In a first step, baseline values were assessed (T0 = baseline, pre-intervention). Second, all participants received an introductory course in health-oriented sitting, which provided basic information on the problems associated with sedentary behavior and guidance on healthy and unhealthy sitting. Unhealthy sitting can be caused by an inadequate coordination between work equipment and furniture, by unfavorable work tasks with repetitive movements or by sitting at a desk for too many hours each day, with too few breaks and with only occasional changes of posture. Healthy sitting is characterized mainly by a correct arm and leg position (90° angle). It is also important to use the entire seating area of the chair and change the seating position. Finally, it is recommended to take a walk in between (e.g., during lunch break) and stand up to hold shorter meetings or telephone conversations. Participants in the intervention group were also given basic instruction in health-oriented standing positions. For example, it was recommended to change the posture two to three times an hour (between standing and sitting). Standing phases should not take longer than 20–30 min. When standing, an upright posture, slightly bent knees, a slightly upright pelvis, and standing parallel to the hip is important. Even when standing a dynamic posture change was recommended. Both courses were led by two trained psychologists in collaboration with the local Corporate Medical Occupational Health Services of the university. In total, 100 people (78.7%) took part in the healthy sitting course (control: *n* = 45, intervention: *n* = 55) and 55 employees from the intervention group also participated in the healthy standing course. After the course each participant also received a flyer with important information on healthy sitting (and standing). Employees in the intervention group subsequently received an electrically height-adjustable SSD, which the employees were allowed to continue using after the trial has ended.

Over a period of six months, variables were assessed monthly on a self-reported basis (T1 to T6). The present study augmented this data by asking the same participants for the same information 24 months later (i.e., 18 months after the T6 measurement; T24). To ensure anonymity, each participant was assigned an individual code, which was used throughout the study. Each subject received a €5 voucher for participating in the study.

Measures.

### Musculoskeletal complaints

We measured the intensity and prevalence of MSC on a monthly basis (T0 to T6) and at the 24-month follow-up (T24) using six items each for intensity and prevalence, based on the Stress Report Germany [42]. Participants were asked how strongly and how often (i.e., intensity and prevalence) over the past month they had experienced any of the following specific complaints during a working day: pain in the lower back, pain in the neck or shoulder area, pain in the arms or hands, pain in the hips, pain in the knees, and pain in the legs or feet. The intensity of complaints was assessed on a five-point scale, ranging from *no impairment* (1) to *very severe impairment* (5), and the prevalence of complaints was assessed on a five-point scale, ranging from *never* (1) to *very often* (5). Cronbach’s alpha was 0.70 (T0–T6) and 0.75 (T24) for MSC intensity, and 0.67 (T0–T6) and 0.75 (T24) for MSC prevalence. For the analyses we used mean scores for both MSC intensity as well as MSC prevalence according to [42]. The ratings of each body region in terms of intensity and prevalence of symptoms were each aggregated to a mean score, yielding one mean score for MSC intensity and one mean score for MSC prevalence. Individual analyses of single body regions were not performed.

### Activation

Activation (vitality and vigilance) was measured monthly (T0–T6) and at the 24-month follow-up (T24); for both vitality and vigilance four items were used from the Basler Mental State Scale [26]. The semantic differential measure consisted of pairs of polar-opposite adjectives describing different experiences of activation and used a five-point scale from 1 (s*trongly agree*) to 5 (*strongly agree*). Example items are “During the last weeks while or after doing desk work I felt weakened vs. strengthened” for vitality and “… inattentive vs. attentive” for vigilance, where higher values represent high activation levels and lower values represent lower activation levels. Cronbach’s alpha ranged between 0.93 (T0–T6) and 0.87 (T24) for vitality, and between 0.96 (T0–T6) and 0.91 (T24) for vigilance.

### Data analysis

The dataset contained a total of 982 observations (i.e., 127 subjects × 7 points in time and 93 subjects from the follow-up). Unless otherwise stated, in all analyses we used a Bayesian estimator to avoid reliance on a large-sample theory and normal distribution assumptions, thus producing more accurate estimates [43, 44]. Bayesian analyses were conducted in Mplus 8.4 [45] using two Markov chain Monte Carlo chains and a minimum of 20,000 iterations and a maximum of 100,000 iterations, with every 20th sample—to avoid autocorrelation—stored and used for posterior inference. We used the default Gibbs PX1 setting for the Markov chain Monte Carlo algorithm to generate the posterior distribution of the parameters, and the default point estimate was the median [45]. All models were evaluated following current standards [45] for absolute and comparative fit, including the posterior predictive p-value (PPP), which should exceed 0.05, the 95% confidence interval for the difference between observed and replicated χ²-values (PPC-CI), which should include zero, the RMSEA, which should be < 0.06, the CFI and TLI, which should be > 0.95, and the DIC and BIC, where lower values indicate better comparative fit.

Before evaluating model fit, we also checked the convergence of model estimation. The convergence behavior of the Markov chains and the quality of the target posterior distribution for each parameter of the simulation was fulfilled, which was indicated by (1) the convergence criterion (PSR < 1.004), the highest PSR was as low as 13 (range 2 to 13) after 20,000 iterations and does not bounce over more iterations; (2) trace plots that revealed that each of the two chains burn-in and posterior distribution has reached equilibrium quickly and mixes rapidly; (3) autocorrelation plots between parameter estimates lagged by iterations revealed a desirable small autocorrelation (e.g., 0.1 or less); (4) a density function that indicates a good simulation of the posterior distribution (for details, see [46]).

We examined Hypotheses 1 and 2 with structural equational analyses. Here, we used the manifest values at T0 (baseline) and T24 (follow-up) as single indicators for latent values to account for random and measurement error [47]. Subsequently, we used this latent value for T0 and the dichotomous variable treatment (0 = control group; 1 = treatment group) as predictors for the latent value at T24, thus using the latent residualized change approach [48].

To examine Hypotheses 3 and 4 we used the latent change and latent acceleration framework [49], a conceptually powerful tool for studying different aspects of change (i.e., growth, velocity, and acceleration) within a single model. Based on growth modeling and latent change score modeling, this approach allows one to study between-person differences in the rate of change (i.e., velocity) and change in velocity over time (i.e., acceleration), while also considering autoregressive relationships. Positive velocity scores indicate high levels of positive growth (i.e., values increase with time) and positive acceleration scores indicate high levels of growth in velocity (i.e., velocity increases with time). For measures of MSC this means that a negative velocity score indicates a decrease in symptomatology over time, and for measures of activation a positive velocity indicates an increase in activation over time.

We compared fit of quadratic and cubic acceleration models for each variable (intensity/prevalence of MSC, vigilance and vitality) using the first six measurement points (i.e., T0–T5). The fit indices CFI, TLI, and RMSEA indicate that cubic models perform substantially better, while the BIC and DIC indices are only slightly higher than for the quadratic model. For this reason, we considered the cubic models to examine Hypotheses 3 and 4. Next, we analyzed the regressions for each outcome at T6 and T24 that was predicted by the initial velocity and initial acceleration using a residualized change approach that accounts for baseline differences (see [48]). Thus, the T0 score of the outcome is added as an additional predictor to the regression. Hereby, initial velocity technically refers to the first latent change parameter of observations (i.e., first latent difference score of observations) and initial acceleration technically refers to the first latent change parameter of velocity parameters (i.e., latent change scores of observations). The Mplus input and a path diagram for the latent acceleration model are shown in Appendix B and C, respectively.

## Results

In total, 127 (n = 58 intervention group; n = 69 control group) participants completed the initial survey (T0) and 98 participants completed the follow-up survey (T24). One person in the intervention group stated that he/she no longer uses the SSD and one person in the control group indicated that he/she now uses an SSD. Both were excluded from subsequent analyses. In addition, three participants were removed because their codes could not be matched to the original data; this left us with a final sample of 93 (73.2% of the original sample) of which 47 belonged to intervention group and 46 belonged to the control group. More detailed information about the allocation ratio can be found in the flow diagram in Appendix A. Using panel data to analyze longitudinal models of health carries a risk that the result may be contaminated by a bias associated with longitudinal non-response, which is a serious problem for longitudinal models in health research [50, 51]. To test for attrition bias, we performed χ^2^- and *t*-tests comparing baseline characteristics for respondents vs. non-respondents. According to standards for reporting on attrition in randomized controlled trials [52], we found no evidence of differential attrition. Little’s [53] MCAR-test indicated that missing data (22.9% at T24) were missing completely at random (χ² = 1728.90, *df* = 1664, *ns.*).

**Fig. 1:**
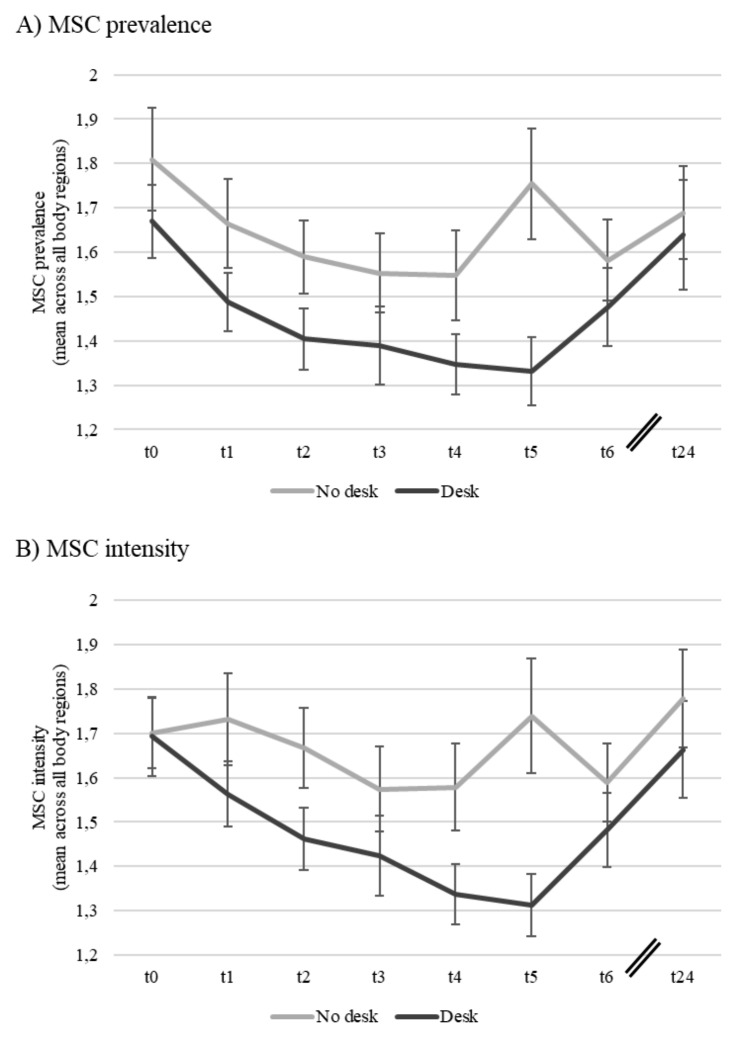
*Mean Scores of Musculoskeletal Complaints (MSC) Across all Body Regions for both Groups with Standard Errors of Means (Note:The first wave (t0) represents the baseline prior to implementing the intervention. The last wave (t24) represents the follow-up measurement 24 months after the beginning of the intervention. Activation scores were measured on a scale from 1 to 5.)*

**Fig. 2:**
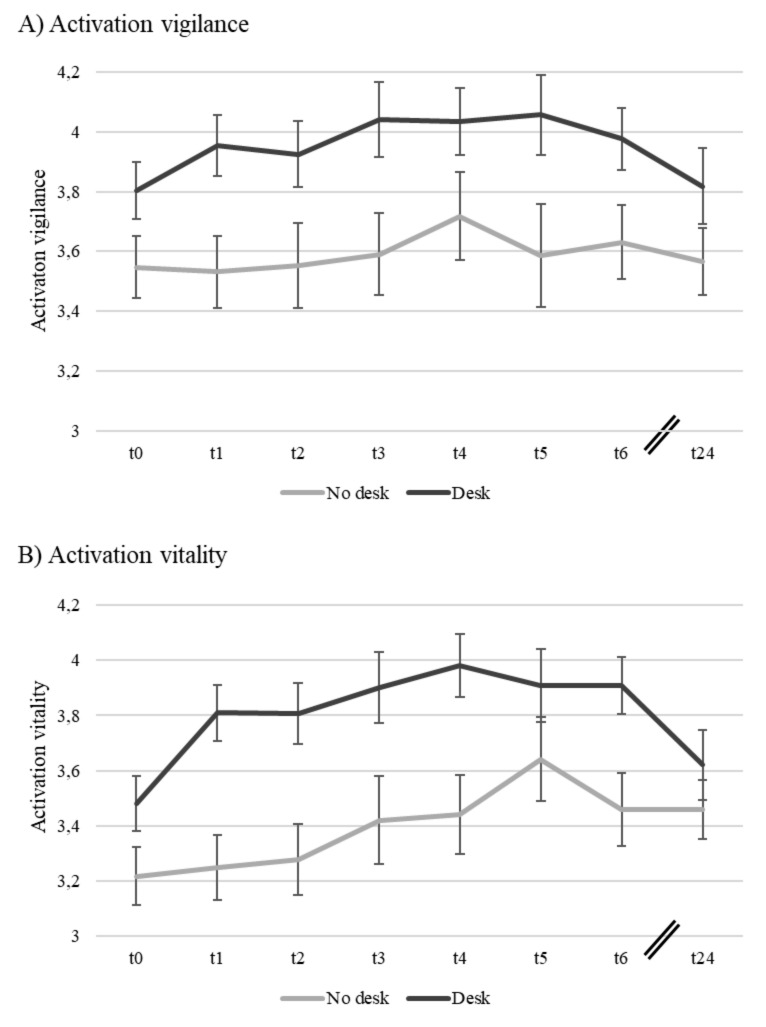
*Mean Scores of Activation for both Groups with Standard Errors of Means(Note:The first wave (t0) represents the baseline prior to implementing the intervention. The last wave (t24) represents the follow-up measurement 24 months after the beginning of the intervention. Activation scores were measured on a scale from 1 to 5.)*

Means, standard deviations and bivariate correlations are presented in Table 1. Hypothesis 1, which predicted that employees in the SSD intervention group would show a long-term decrease in prevalence and intensity of MSC compared to employees in the control group, was rejected. As shown in Table 2 there was no significant prediction of follow-up scores by treatment controlled for their respective baseline levels at T0. Model fits were excellent for both intensity (PPP = 0.528, PPC CI = -11.50, 10.98, RMSEA = 0.00, CFI = 1.00, TLI = 1.00) and prevalence (PPP = 0.440, PPC CI = -10.85, 11.83, RMSEA = 0.00, CFI = 1.00, TLI = 1.00) of MSC. Similarly, there was no support for Hypothesis 2, which predicted that employees working at SSDs would show a long-term increase in activation (i.e., vigilance and vitality) compared to those in the control group. Model fits for both vigilance (PPP = 0.284, PPC CI = -8.51, 15.15, RMSEA = 0.10, CFI = 0.95, TLI = 0.87) as well as vitality (PPP = 0.294, PPC CI = -8.37, 14.25, RMSEA = 0.06, CFI = 0.93, TLI = 0.91) were excellent. The means and standard errors of mean for prevalence and intensity of MSC for both groups are shown in Fig. 1 A and B, respectively. The means and standard errors of mean for vigilance and vitality for both groups are shown in Fig. 2 A and B, respectively.

**Table 1:** *Descriptive Statistics, Reliabilities, and Correlations*

	Variable	*M* (*SD*)	1	2	3	4	5
1.	Intervention^a^	−	−				
2.	MSC – intensity^b^	1.51 (0.49)	− 0.15	(0.71)	**0.88**	**− 0.20**	**− 0.37**
3.	MSC – prevalence^b^	1.50 (0.51)	− 0.16	**0.98**	(0.68)	**− 0.18**	**− 0.34**
4.	Vigilance^c^	3.95 (0.65)	0.26	− 0.27	− 0.24	(0.93)	**0.77**
5.	Vitality^c^	3.74 (0.67)	**0.32**	**− 0.49**	**− 0.45**	**0.89**	(0.90)

*Note.*^a^ Intervention: 0 = control, 1 = treatment. MSC = musculoskeletal complaints

^b^ Mean scores across all body regions were aggregated over the measurement points. ^c^ Mean scores were aggregated over the measurement points. The lower half of the table shows between-subject correlations (*N* = 127); the upper half shows within-subject correlations (*N* = 752 to 756). Numbers in parentheses are Cronbach’s alpha reliabilities where appropriate. Coefficients in bold face indicate statistical significance at *p <* .05 (two-tailed)

**Table 2:** *Coefficients from Latent Residualized Change Regression Models Predicting Health at the 24-Month Follow-up*

Predictors	β *(PSD)*	90% CI	*p*
*Model 1 – Criterion: Intensity of MSC*			
Baseline (T0)	0.78 (0.14)	0.54, 0.97	0.001
Intervention (0 = control, 1 = treatment)	-0.02 (0.11)	-0.21, 0.16	0.424
*Model 2 - Criterion: Prevalence of MSC*			
Baseline (T0)	0.73 (0.17)	0.42, 0.96	0.001
Intervention (0 = control, 1 = treatment)	0.03 (0.13)	-0.19, 0.23	0.414
*Model 3 - Criterion: Vigilance*			
Baseline (T0)	0.77 (0.15)	0.50, 0.97	0.001
Intervention (0 = control, 1 = treatment)	-0.10 (0.12)	-0.30, 0.09	0.203
*Model 4 - Criterion: Vitality*			
Baseline (T0)	0.66 (0.20)	0.31, 0.95	0.002
Intervention (0 = control, 1 = treatment)	-0.06 (0.14)	-0.29, 0.16	0.324

*Note. N* = 93. MSC = musculoskeletal complaints. β = standardized path coefficient. PPP = Posterior Predictive P-value. *PSD* = Posterior Standard Deviation. CI = credibility interval. Model fit: Model 1: PPP = 0.528, PPC CI = -11.50, 10.98, RMSEA = 0.00, CFI = 1.00, TLI = 1.00; Model 2: PPP = 0.440, PPC CI = -10.85, 11.83, RMSEA = 0.00, CFI = 1.00, TLI = 1.00; Model 3: PPP = 0.284, PPC CI = -8.51, 15.15, RMSEA = 0.10, CFI = 0.95, TLI = 0.87; Model 4: PPP = 0.294, PPC CI = -8.37, 14.25, RMSEA = 0.06, CFI = 0.93, TLI = 0.91.

### Hypothesis

a predicted that employees in the SSD intervention group with initially high velocity of decrease in MSC show lower medium-term and lower long-term levels of MSC. In partial support of Hypothesis 3a, initial high velocity of decrease in MSC was a statistically significant predictor of lower medium-term levels of intensity and prevalence of MSC (see Table 3). However, no significant long-term relationships were found. Model fit was excellent (PPP = 0.317, PPC CI = -21.56, 36.01, RMSEA = 0.02, CFI = 1.00, TLI = 1.00).

**Table 3:** *Coefficients of Residualized Change Regression Models for Velocity and Acceleration of Health Improvements Predicting Health at the Medium-term and Long-term*

Predictors	Medium-term (6 months)				Long-term (24 months)		
	β *(PSD)*	90% CI	*p*		β *(PSD)*	90% CI	*p*
*Model 1 – Criterion: Intensity of MSC* ^a^							
Velocity	**1.76 (0.90)**	0.515, 3.420	0.012		0.52 (0.96)	-1.162, 1.974	0.282
Acceleration	0.94 (0.87)	-0.237, 2.538	0.098		-0.36 (0.83)	-1.775, 0.955	0.310
*Model 2 – Criterion: Prevalence of MSC* ^a^							
Velocity	**3.05 (1.30)**	1.239, 5.464	0.004		1.90 (1.30)	-0.020, 4.185	0.052
Acceleration	**2.67 (1.28)**	0.859, 5.015	0.010		1.24 (1.33)	-0.711, 3.594	0.144
*Model 3 – Criterion: Vigilance* ^a^							
Velocity	0.89 (1.45)	-1.303, 3.333	0.206		**2.15 (1.65)**	0.342, 5.475	0.030
Acceleration	0.10 (1.50)	-3.103, 2.688	0.462		1.89 (1.69)	-0.055, 5.231	0.054
*Model 4 – Criterion: Vitality* ^a^							
Velocity	0.84 (1.60)	-1.822, 3.295	0.250		1.88 (1.86)	-0.904, 4.959	0.094
Acceleration	0.01 (1.57)	-2.536, 2.504	0.497		1.26 (1.81)	-1.437, 4.253	0.169

*Note. N =* 58. MSC = musculoskeletal complaints. β = standardized regression coefficient. PPP = Posterior Predictive P-value. *PSD* = Posterior Standard Deviation. CI = credibility interval. ^a^ Outcome controlled for its baseline value (T0). Coefficients in bold face indicate that the 90% CI does not contain the zero. Model fit: Model 1: PPP = 0.317, PPC CI = -21.56, 36.01, RMSEA = 0.02, CFI = 1.00, TLI = 1.00; Model 2: PPP = 0.185, PPC CI = -15.63, 42.00, RMSEA = 0.07, CFI = 0.98, TLI = 0.97; Model 3: PPP = 0.233, PPC CI = -17.20, 39.49, RMSEA = 0.05, CFI = 0.97, TLI = 0.97; Model 4: PPP = 0.183, PPC CI = -15.95, 40.83, RMSEA = 0.07, CFI = 0.95, TLI = 0.93.

### Hypothesis

b, which predicted that the initial high velocity of increase in activation during the early phases of the intervention would be predictive of higher medium- and long-term levels of activation received partial support, as we did find a significant positive association between velocity and long-term levels of vigilance. Similarly, model fit can be considered adequate (PPP = 0.185, PPC CI = -15.63, 42.00, RMSEA = 0.07, CFI = 0.98, TLI = 0.97).

### Hypothesis

a predicted that acceleration of decrease in MSC during the early phases of the intervention would predict lower medium- and long-term levels of prevalence and intensity of MSC. The effect was only significant for medium-term levels of prevalence and not for long-term levels of prevalence. For medium- and long-term levels of intensity we found no significant effects. As with the previous models the model fit was excellent (PPP = 0.233, PPC CI = -17.20, 39.49, RMSEA = 0.05, CFI = 0.97, TLI = 0.97).

The results for Hypothesis 4b, which assumed that acceleration of an increase in activation during the early phases of an intervention would predict higher medium- and long-term levels of activation (vigilance and vitality) indicated that acceleration of change had no significant effects on activation. The model fit was adequate (PPP = 0.183, PPC CI = -15.95, 40.83, RMSEA = 0.07, CFI = 0.95, TLI = 0.93).

## Discussion

The present longitudinal randomized controlled trial study examined whether effects of an SSD intervention on employees’ health persist or fade out and whether velocity and acceleration of improvements can predict medium-term and long-term improvements. Because we combined a randomized control trial with a longitudinal study design, we can obtain unbiased estimates of the effect of the intervention across time. Our results also extend the previous lines of occupational health research by contributing to a more general understanding of activity-permissive interventions.

The first main finding of this study is that positive SSD intervention effects might fade out over time. More precisely, we showed that the health effects found on the medium-term level [12] did not persist on the long run. In our study, there was neither a reduction in the prevalence and intensity of MSCs at the 24-month follow-up compared to the baseline nor an improvement of activation in terms of vigilance and vitality. To understand these fade-out effects we examined in a next step whether the velocity and acceleration in improvements can predict medium-term and long-term health improvements. We thus tested whether employees can benefit from a prompt progress at the beginning of the intervention in the medium and long run.

Our findings imply that velocity of the reduction of MSC predicted medium-term improvements in MSC and velocity of the increase of activation also predicted long-term improvements in vigilance. Therefore, employees benefit from a higher velocity in health improvements at the beginning of an intervention both in the medium-term and long-term. We also demonstrated that acceleration of the reduction of MSC predicted medium-term improvements in the prevalence of MSC, implying that employees benefit from a higher acceleration in health improvements at the beginning of an intervention at least in the medium-term. To our knowledge, no previous study has addressed the acceleration of improvements in health within an occupational health intervention. Consistent with research [15, 54] and guiding theory [15, 18] we assume that initial success generates a psychological momentum, leading to maintain beneficial medium- and long-term health effects and preventing fade-out effects. Though obvious, however, the timing of health-related goal attainment has received scant attention in occupational health research, for which various reasons can be advanced. Most studies have used designs involving seldomly more than two waves or captured relatively short periods of time. Therefore, they are often underpowered to detect dynamic effects (e.g., [55]). Our findings suggest future research to consider an intervention as a continuous process that also allows for exploring self-regulatory processes and reciprocal effects of psychological momentum and outcomes, which can take the form of higher structures such as upward or downward spirals [12, 56, 57]. More generally, the results of our study together with meta-analytic evidence [58] imply that it is essential to investigate the long-term effects of interventions.

The findings of our study accordingly also offer practical implications for the economic efficiency, target group specificity, and implementation of SSDs. SSDs are a low-threshold workplace intervention, being easy to use and relatively cost-effective from an employer’s perspective. Even though our results indicate that their effectiveness is limited, and effects might fade out over time, interventions in the workplace can contribute significantly to improve employees’ health over time, and this has a positive effect on productivity as there is less absenteeism through sickness, and other healthcare and intervention costs are lower [59]. However, to sustain these positive effects and prevent them from fading out, there is the need for behavioral transitions in the early phases of the intervention; it is important to encourage and support employees to use the SSDs, particularly in the first few weeks of the intervention because we demonstrated that early velocity and acceleration in health improvements contribute to the persistence of intervention effect and prevent fade-out effects. In addition, organizations should stress to employees the importance of changing one’s posture frequently, especially at the beginning of the intervention. This can be achieved by developing a culture and climate that provides support for managers, occupational services, and frequent occupational health care meetings and health circles (e.g., [60]; see also [61]). Drawing on frameworks and best practice on sustained workplace interventions (e.g., [62]), managers and colleagues should be alert to how well employees are following the program, should be supportive of employees’ health-related behavior, and should influence the behavior of others through model learning. They should also exert social control by using specific behavioral tactics, such as asking individuals about their progress towards their goal and encouraging them to use SSDs daily [30].

Finally, in addition to providing support at the beginning of the intervention, we recommend that organizations set up a follow-up training after, for example, 24 months to refocus employees on the benefits and functionality of SSDs and encourage continued use to prevent fade-out effects.

### Limitations and future research

Beyond the theoretical and practical contributions of our study, it has five main limitations, which suggest important directions for future research. First, it is not clear whether we selected a sampling frame (i.e., a sampling interval and sampling rate) that aligns with the inherent dynamic of the variables. Since there is no appropriate psychological theory to guide us on this, other settings might have yielded stronger effects [63]. More research is needed to understand if and how positive effects persist over longer periods of time and how fade-out effects can be prevented. Second, even though self-reports of health status are widely considered to be valid indicators of objective health status and longevity, they are also potentially biased [64]. Examination of our hypotheses using multiple assessment methods, including objective methods could help address these limitations. With this in mind, the results are limited by the lack of objective desk usage data. Thus, conclusions about positive findings based on the use of the SSDs are limited, since, for example, no objective dose-response relationship can be determined. Future research should therefore attempt to establish a link between the objective frequency of usage of the SSDs and beneficial health outcomes to be able to investigate more precisely whether, for example, there are thresholds of usage above which a positive trend is indicated.

Third, our results may be limited using composite scores for MSC intensity and MSC prevalence that were averaged across all body regions rather than exclusively examining different body regions. However, in accordance to [42], we used the composite scores because they have higher content validity and cover different body regions, all of which are expected to be affected by the use of SSDs. Nevertheless, future research should investigate whether certain body regions respond better to this type of intervention than others.

Fourth, although numerous meta-analyses have considered self-regulation to be a primary mechanism in interventions designed to change health-related behavior, studies have usually failed to examine the underlying processes or have focused narrowly on self-efficacy, frequency of self-monitoring or cognitive bias [65]. Future studies could consider the processes behind the psychological momentum driving the effects of health interventions because they serve as potential starting points for interventions aimed at promoting behavioral change. Theory suggests that the positive relationship between velocity and a decrease in the medium-term intensity of MSC is mediated by the effort made by an individual to use the SSD (see [30]). At a cognitive level, executive functions such as attentional control, cognitive inhibition, and inhibitory control are involved in monitoring behaviors and help individuals to achieve their goals [66]. Individuals who are repeatedly asked to assess their health might become more sensitive to their own health status, which serves as a feedback and they may progressively engage in health-related behavior. Conversely, deficits in executive function—for example, when individuals perceive the processes to be beyond their control—impair motivation to engage in the self-regulation needed to attain one’s goals over time.

Finally, we also advocate future research that investigates to what extent the effects of an SSD intervention are specific to the individual [67] and type of workplace [68] and whether there are subgroups of individuals or working in home office showing different patterns of response. For example, there may be a class of slow responders, who show low velocity in health improvement at the beginning, but once they adapt to the SSD, their psychophysical conditions strongly improve. Person-centered and person-specific analyses might identify distinct unobserved groups (i.e., latent classes) of individuals who share self-regulation profiles over time.

## Conclusion

Long-term effects of occupational health interventions play an important role in improving employees’ health and quality of life, but can also help to reduce the economic risks of absence from work due to sickness, loss of productivity, and reduced ability to work. We have demonstrated that implementing SSDs can improve employees’ health, although this effect faded out two years after the intervention has started. More importantly, our results suggest that this fade-out could be prevented by increasing the velocity and—to some extend—acceleration in health improvements at the beginning of the intervention, implying that the start of an intervention represents a sensitive period for beneficial effects. In sum, this research adds to our knowledge of the long-term effects of activity-permissive interventions and offers insights into the role of velocity and acceleration in health improvement.

## Electronic supplementary material

Below is the link to the electronic supplementary material.

Supplementary Material 1

Supplementary Material 2

Supplementary Material 3

## Data Availability

The datasets used and/or analyzed during the current study are available from the corresponding author on reasonable request.
