# Supplementary material for: Longitudinal Effects of a sit-stand desk intervention - persistence, Fade-Out, and psychological momentum: a Randomized Controlled Trial
Source: BMC Psychol. 2022 Nov 2;10:246. doi: 10.1186/s40359-022-00948-9 (PMC9632028; doi:10.1186/s40359-022-00948-9)
Supplement: Supplementary file 1 — Supplementary Material 1 [file 40359_2022_948_MOESM1_ESM.docx]

## Appendix B

*Mplus Input for the Latent Acceleration Model*

| TITLE: Latent acceleration model; |
| --- |
|  |
| USEVARIABLES ARE |
|  |
| y1 y2 y3 y4 y5 y6 y7 y24; |
|  |
| *! y1 refers to the baseline measurement* |
| *! y2-y7 refer to the first six measurements* |
| *! y24 refers to the follow-up measurement* |
|  |
| ANALYSIS: |
|  |
| *! use Bayes estimator for analyses* |
| ESTIMATOR = BAYES; |
| BITER = 100000 (20000); |
| THIN = 20; |
|  |
| MODEL: |
|  |
| *! fix error terms* |
|  |
| [y1-y6@0]; |
| y1-y6*1; |
|  |
| *! define latent single indicators* |
|  |
| ly1 BY y1@1; |
| ly2 BY y2@1; |
| ly3 BY y3@1; |
| ly4 BY y4@1; |
| ly5 BY y5@1; |
| ly6 BY y6@1; |
|  |
| *! define autocorrelations* |
|  |
| ly2 ON ly1@1; |
| ly3 ON ly2@1; |
| ly4 ON ly3@1; |
| ly5 ON ly4@1; |
| ly6 ON ly5@1; |
|  |
| *! define latent velocity scores (i.e., difference scores of first order)* |
|  |
| dy2 BY ly2@1; |
| dy3 BY ly3@1; |
| dy4 BY ly4@1; |
| dy5 BY ly5@1; |
| dy6 BY ly6@1; |
|  |
| *! define autocorrelations of the velocity scores* |
|  |
| dy3 ON dy2@1; |
| dy4 ON dy3@1; |
| dy5 ON dy4@1; |
| dy6 ON dy5@1; |
|  |
| *! define latent acceleration scores (i.e., difference scores of second order)* |
|  |
| ddy3 BY dy3@1; |
| ddy4 BY dy4@1; |
| ddy5 BY dy5@1; |
| ddy6 BY dy6@1; |
|  |
| *! define latent variables corresponding to intercept, linear, quadratic, and cubic slopes, respectively* |
|  |
| eta1 BY ly1@1; |
| eta2 BY dy2@1; |
| eta3 BY dy2@1 |
| ddy3@2 |
| ddy4@2 |
| ddy5@2 |
| ddy6@2; |
|  |
| eta4 BY dy2@1 |
| ddy3@6 |
| ddy4@12 |
| ddy5@18 |
| ddy6@24; |
| eta1; |
| eta2; |
| eta3; |
| eta4; |
| [eta1 eta2 eta3 eta4]; |
| eta1 WITH eta2*0; |
| eta1 WITH eta3*0; |
| eta1 WITH eta4*0; |
| eta2 WITH eta3*0; |
| eta2 WITH eta4*0; |
| eta3 WITH eta4*0; |
|  |
| ly1-ly6@0; |
| dy2-dy6@0; |
| ddy3-ddy6@0; |
|  |
| *! regressions for mid- and long-term outcomes controlled for baseline values* |
|  |
| y7 on dy2 ddy3 y1; |
| y24 on dy2 ddy3 y1; |
|  |
| *! standardized output* |
|  |
| OUTPUT: STDYX; |
