# Supplementary material for: Longitudinal Effects of a sit-stand desk intervention - persistence, Fade-Out, and psychological momentum: a Randomized Controlled Trial
Source: BMC Psychol. 2022 Nov 2;10:246. doi: 10.1186/s40359-022-00948-9 (PMC9632028; doi:10.1186/s40359-022-00948-9)
Supplement: Supplementary file 2 — Supplementary Material 2 [file 40359_2022_948_MOESM2_ESM.docx]

## Appendix C

*Path Diagram for the Latent Acceleration Model*


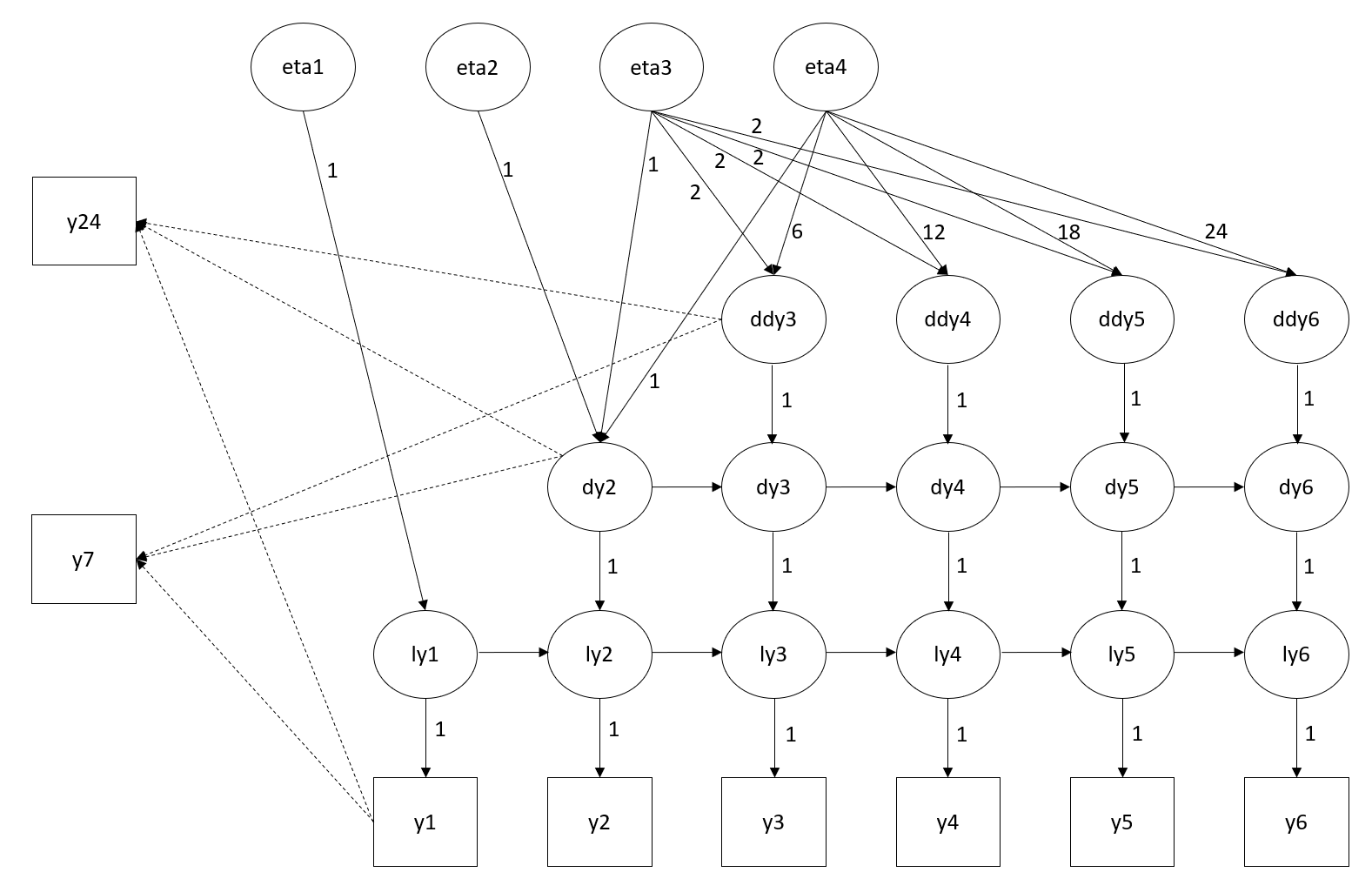


*Note. y1 refers to the baseline measurement, y2-y7 refer to the first six measurements, and y24 refers to the follow-up measurement.*
