# Supplementary material for: Longitudinal Effects of a sit-stand desk intervention - persistence, Fade-Out, and psychological momentum: a Randomized Controlled Trial
Source: BMC Psychol. 2022 Nov 2;10:246. doi: 10.1186/s40359-022-00948-9 (PMC9632028; doi:10.1186/s40359-022-00948-9)

## Appendix A

*Flow Diagram of the Progress Through the Phases of our Randomized Controlled Trial of two Groups (Enrollment, Allocation, Follow-up, and Analysis)*


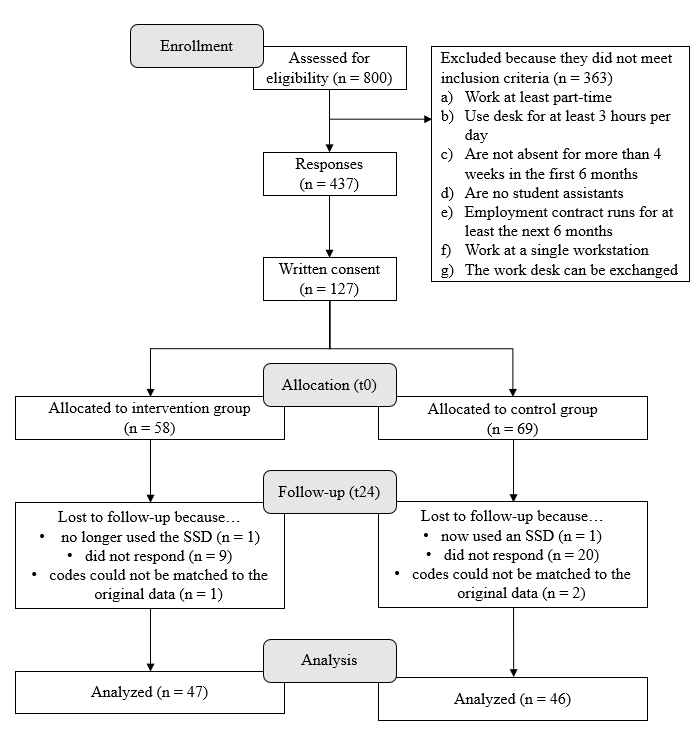

Supplement: Supplementary file 3 — Supplementary Material 3 [file 40359_2022_948_MOESM3_ESM.docx]
